# An Intramedullary Approach to the Management of Pathological Distal Femur Fracture in Genochondromatosis: A Case Report

**DOI:** 10.7759/cureus.86340

**Published:** 2025-06-19

**Authors:** Apoorva Kabra, Nitish J Jyoti, Samarth Mittal, Vivek Trikha

**Affiliations:** 1 Department of Orthopaedics, All India Institute of Medical Sciences, New Delhi, New Delhi, IND

**Keywords:** dfn, distal femur nailing, genochondromatosis, multiple enchondromas, pathological fracture, retrograde nailing

## Abstract

Genochondromatosis is a rare disorder characterized by symmetrical enchondromas, most commonly affecting the knee joint. Although benign, the presence of cartilaginous lesions weakens the bone, predisposing individuals to pathological fractures that present significant management challenges due to altered anatomy, limited treatment guidelines, and restricted implant options. We present the first documented case of managing a pathological distal femur fracture in a 22-year-old male patient with genochondromatosis. The patient presented with an inability to bear weight following a fall. Clinical and radiological evaluation revealed multiple metaphyseal lesions involving the femur, tibia, humerus, clavicle, radius, and ulna, consistent with genochondromatosis. A family history of similar skeletal abnormalities suggested a hereditary pattern. Due to the abnormal morphology of the distal femur, conventional extramedullary fixation was unsuitable. An intramedullary implant was selected for its biomechanical benefits, offering comprehensive stabilization across the femur and promoting optimal healing. Surgical challenges included the flared distal fragment and the absence of normal anatomical landmarks. Postoperative follow-up showed a progressive union of the fracture. The patient was allowed full weight bearing at three months and achieved complete functional recovery by 12 months. This case demonstrates the effective use of an intramedullary implant in a complex pathological fracture associated with genochondromatosis and underscores the importance of individualized pre-operative planning and fundamental fracture management principles in the setting of benign skeletal dysplasias.

## Introduction

Genochondromatosis is a rare form of multiple enchondromatosis. Unlike Ollier's disease, Maffucci syndrome, and spondyloenchondrodysplasia, genochondromatosis presents as symmetrical enchondromas throughout the body, mainly affecting the knee joint. Patients exhibit normal stature and no discernible bony deformities [1].

These swellings do not cause pain or disrupt bodily functions, allowing patients to live with minimal interruption. There is no association with the vertebral column, and no malignant transformation has been documented [2]. However, the seemingly innocuous nature of these swellings belies a significant risk: the weak cartilaginous regions, encased within bone at the extremities of long bones, render individuals susceptible to pathological fractures. These fractures present a unique challenge in the realm of orthopedic management, which has never been described previously in these subsets of patients. It is imperative to recognize that the management of pathological fractures in genochondromatosis mirrors the approaches taken where benign tumors or tumor-like growths compromise bone integrity. The patient has provided written consent for publishing the case details in scientific literature.

## Case presentation

Clinical findings

**Figure 1:**
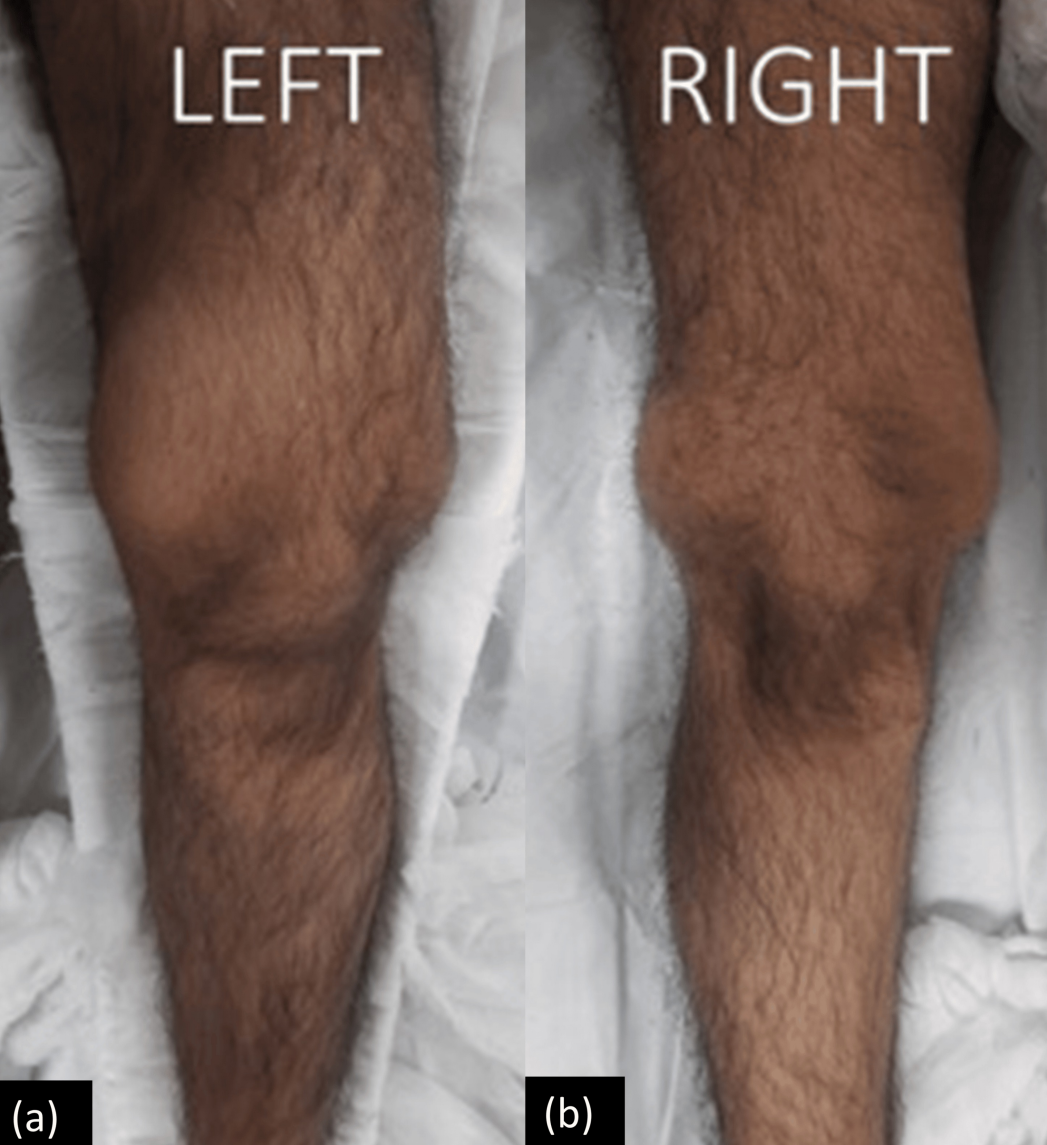
(a) Fusiform swelling on the left distal thigh and knee; (b) a firm swelling was also noted on the right side in the same region.

**Figure 2:**
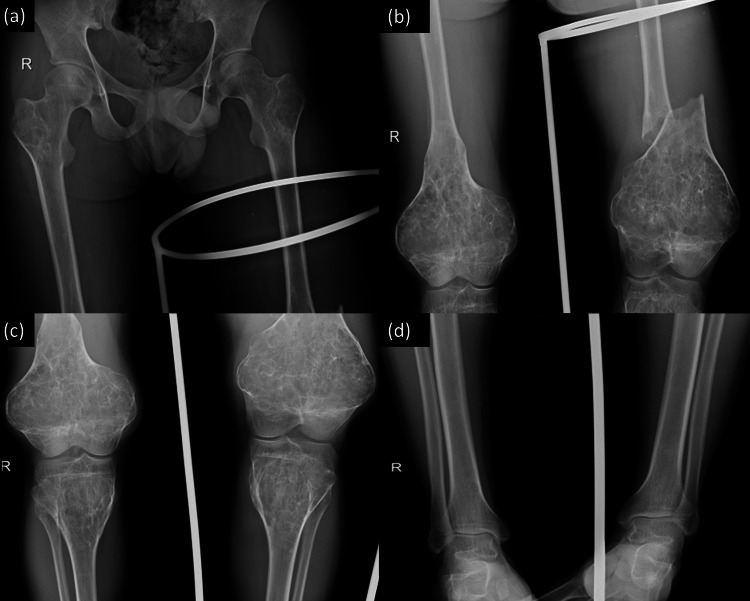
X-rays showing a loss of cortical continuity in the left distal femur and significant flask-like enlargements in the metaphyseal part of both distal femurs and proximal tibia, featuring numerous septations and thinned cortices.

A 22-year-old male patient arrived at our emergency department after a fall, unable to stand or walk, with his left lower limb splinted. Examination revealed a fusiform swelling from the upper knee to the lower thigh, with tenderness (Figure 1). Given the moderate force of the injury and this unusual swelling, we suspected a possible underlying pathological condition. A smaller, non-tender swelling was also observed around the right knee joint, allowing complete motion (0°-130°). Comprehensive imaging revealed a loss of cortical continuity in the left distal femur and significant enlargement in the metaphyseal part of both femurs, with similar lesions in the proximal tibia and femur (Figure 2).

In the emergency department, we immobilized the affected limb, provided intravenous analgesics, administered oxygen, and delivered intravenous fluids. The patient remained stable, with no other major injuries detected.

Three out of his five siblings and his mother had experienced similar swellings. The familial connection, combined with the initial X-ray findings and the mother's healthy life into her middle age, suggested a benign, self-limiting nature of the illness. The sharply defined margins, narrow zone of transition, and absence of significant bone destruction or periosteal reaction further supported this. The patient was a known case of genochondromatosis.

Diagnostic assessment

**Figure 3:**
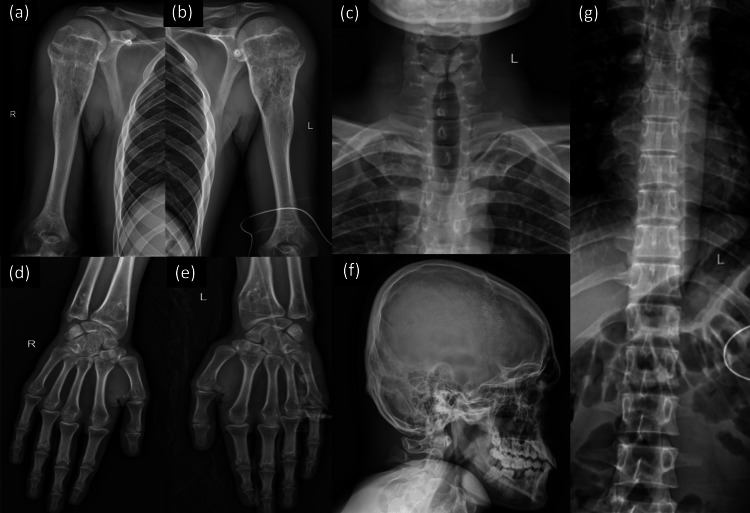
Skeletal survey showing expansile, metaphyseal lesions with chondroid calcification in the proximal humerus (a,b), medial ends of the clavicle (c), and distal radius and ulna (d,e) with no involvement of the skull and vertebral column (f,g).

The skeletal survey revealed a symphony of symmetric, lytic, and expansile metaphyseal lesions with chondroid calcification. These abnormalities were not confined to the femurs but also manifested in the proximal humerus, medial ends of the clavicle, and the distal radius and ulna (Figure 3). No lesions were detected in the skull or vertebral column. To further refine our understanding, bilateral CT scans of the entire femur were performed, which served as a template for fracture fixation. All blood parameters fell within the confines of normalcy. Armed with this detailed knowledge, we were able to not only confirm the diagnosis but also tailor our management strategy with precision.

Differential diagnoses

**Figure 4:**
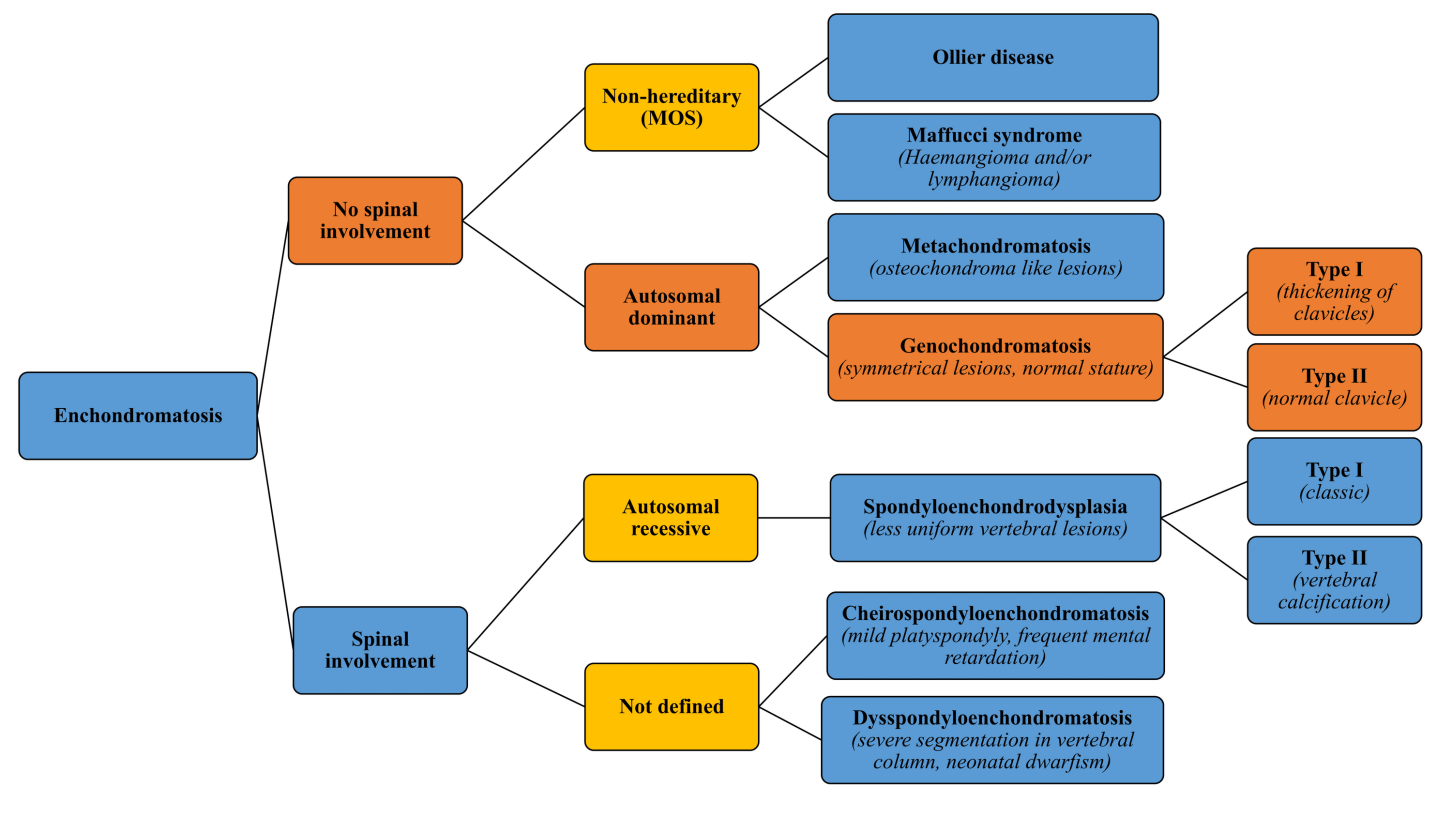
Classification of the various types of enchondromatosis The flowchart is self generated by the authors and requires no copyright permission. Source: Reference [4]

The radiological spectrum of such patients who present with multiple chondroid lesions includes various described pathologies like Maffucci syndrome, Ollier’s disease, genochondromatosis, spondyloenchondrodysplasia, metachondromatosis, and enchondromatosis with 2-glutaric aciduria [3]. In the intricate landscape of enchondromatosis, primary classification pivots on spinal involvement, a critical factor in distinguishing its variants (Figure 4) [4]. Notably, in this case, the absence of spinal involvement coupled with the disease's hereditary nature led us to a definitive diagnosis: genochondromatosis, which was further categorized as type 1 due to the presence of lesions in the medial end of the clavicle (Figure 3), a rare yet significant detail [2,5,6].

Surgical intervention

Treating distal femur fractures, especially in unique cases like this, demands a nuanced approach. Open reduction and internal fixation using anatomical locking plates are most commonly employed in the management of these fractures [7]. However, in our patient's case, the altered anatomy of the distal femur posed a challenge, rendering extramedullary implants unsuitable. This, coupled with concerns about increased blood loss due to the pathological nature of the lesion, doubtful screw stability in the lytic zone of the distal femur, and potential delays in rehabilitation and weight-bearing, made this approach less favorable [8].

Considering these complexities, an intramedullary implant in the form of a distal femur nail (retrograde nail) emerged as the more viable option. This choice was rooted in its biomechanical advantages, offering relative stability, weight-sharing capabilities, and the ability to span a larger portion of the femur, reducing the risks of refracture and stress risers [9].

**Figure 5:**
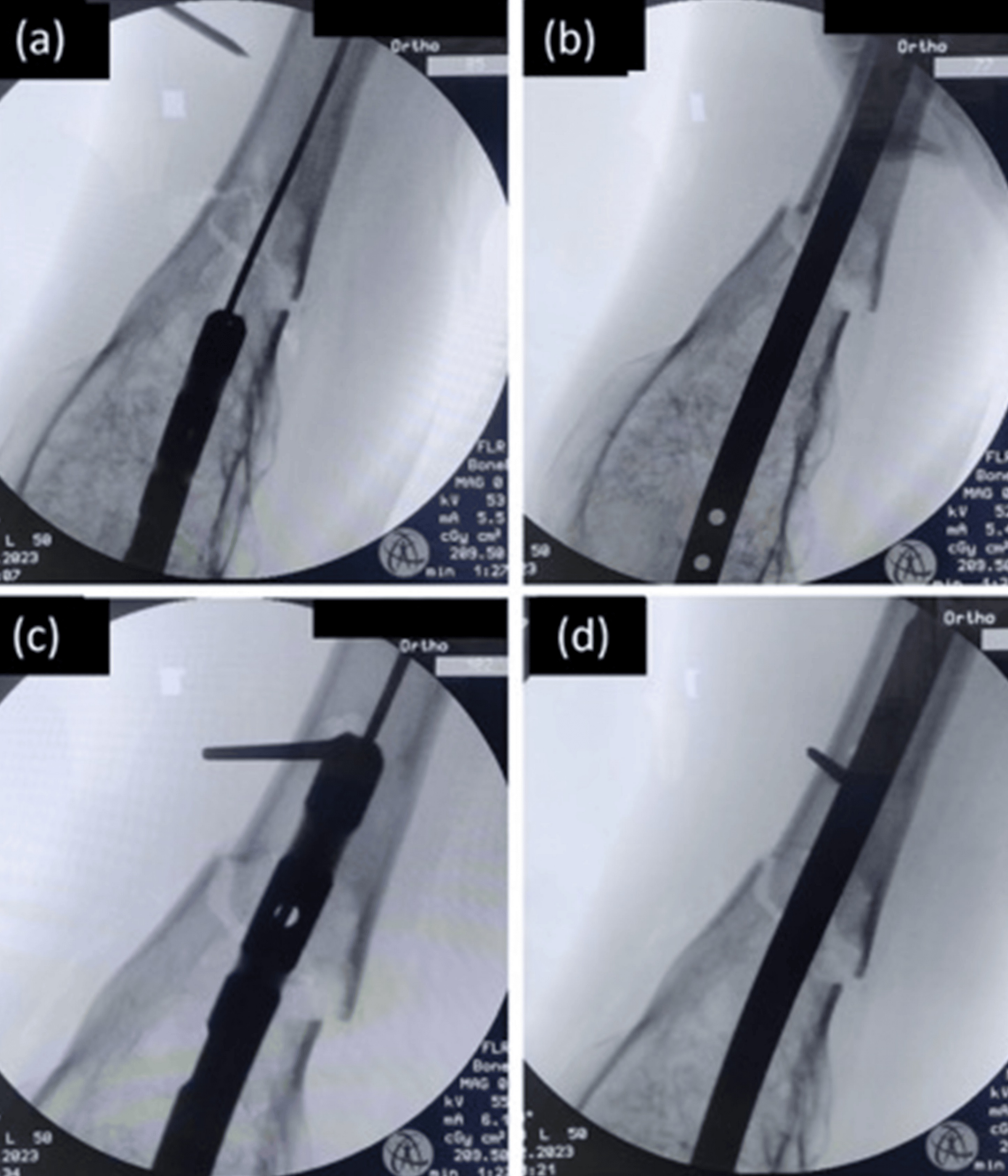
(a,b) Loss of fracture alignment on passage of nail; (c,d) poller pin placed, which prevents this loss of alignment at the fracture site on passage of the nail.

Intramedullary implants typically aid in maintaining femoral fracture reduction by providing alignment between fractured fragments. However, in our patient, the large flared distal fragment complicated this conventional approach. To address this, a poller (blocking) pin was strategically used during surgery. This pin was instrumental in guiding the nail through a specific pathway in the femoral canal, reducing the degree of angulation (Figure 5) [10].

**Figure 6:**
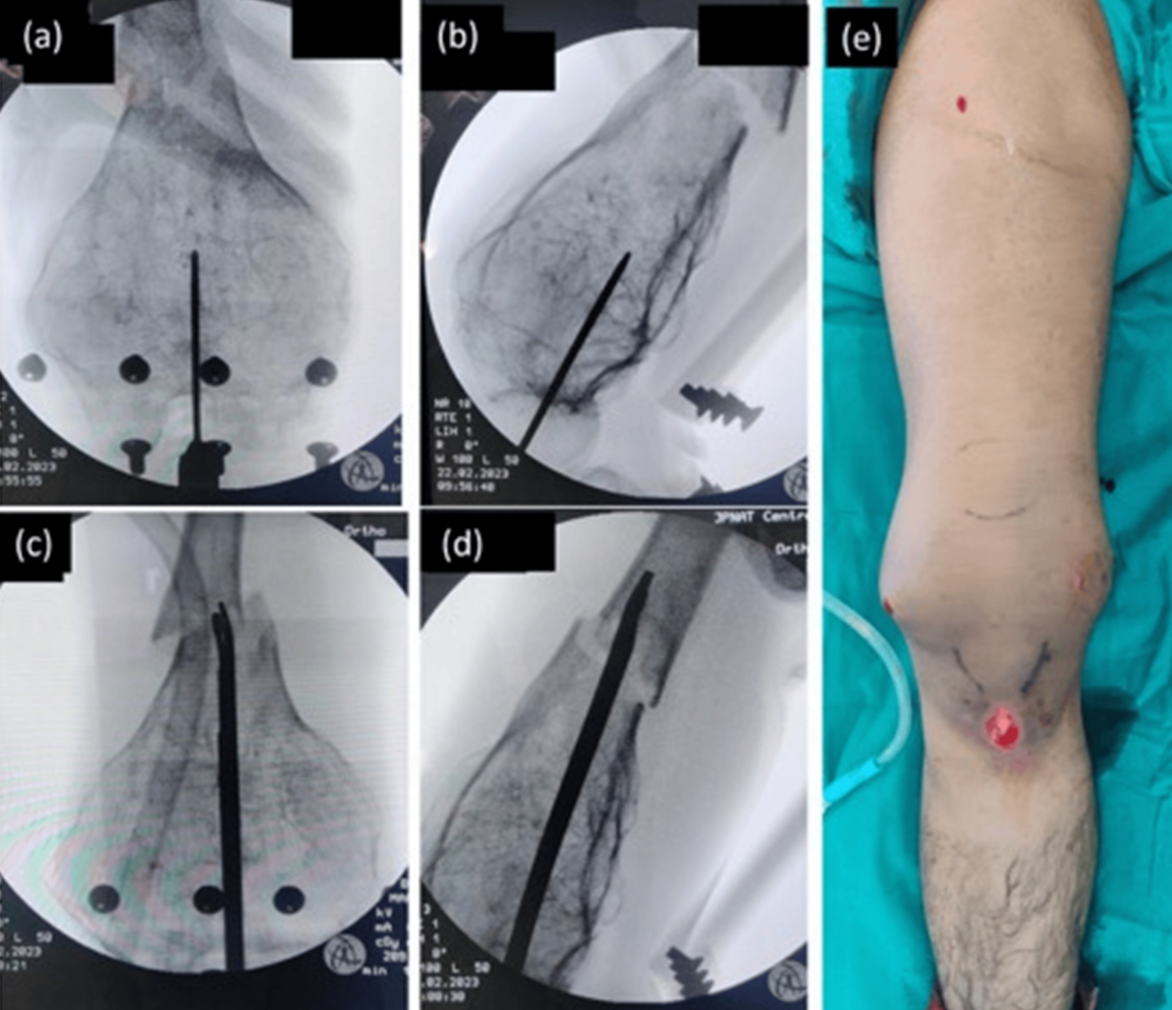
(a,b) Intraoperative images showing retrograde entry to the femoral canal being created using a guidewire keeping the entry point slightly posterior and in the middle of the flared distal femur; (c,d) use of a finger reducer tool to pass the guidewire across the fracture site; (e) surgery completed using a minimally invasive approach, without opening the fracture site.

Additionally, the loss of usual anatomical landmarks, like Blumensaat’s line and the actual center of the femur due to asymmetrical expansion, presented a further hurdle. Relying on clinical judgment and intraoperative assistance from specialized instruments, such as the finger reducer for passing the long guidewire into the proximal segment and the poller screw to maintain reduction lost during nail insertion, was crucial (Figure 6). Moreover, dealing with the mismatch between available interlocking screw sizes and the diameter of the flared fragment required strategic placement of the longest available screws, ensuring a secure grip in the bone [11].

Adhering to the foundational principles of trauma care, we successfully achieved satisfactory fixation of this pathological fracture through a minimally invasive approach (Figure 6). Postoperatively, the patient commenced knee range of motion exercises immediately and was allowed protected weight-bearing using a height-adjustable walker. This tailored and meticulous approach not only addressed the immediate fracture but also navigated the intricacies of the patient's unique pathology, ensuring a comprehensive and effective treatment outcome.

Follow-up and outcomes

Given the scarcity of literature addressing pathological fractures in genochondromatosis, a critical gap existed regarding the anticipated bone healing trajectory-whether it would align with conventional fractures or deviate from the norm. To bridge this knowledge gap, the patient underwent meticulous monitoring at regular intervals post-surgery, including assessments at two weeks, six weeks, three months, six months, and one year.

At the two-week mark, staples were removed. The patient exhibited a range of motion, reaching 0°-90° during this period. By the six-week follow-up, radiographs revealed the presence of callus formation, indicative of the ongoing healing process. Importantly, the fracture maintained its alignment, affirming the efficacy of the treatment approach.

With the assurance of maintained alignment and the presence of callus formation, the patient was permitted to bear full weight on the affected limb over the next six weeks. During this period, the use of assistive devices was withdrawn.

**Figure 7:**
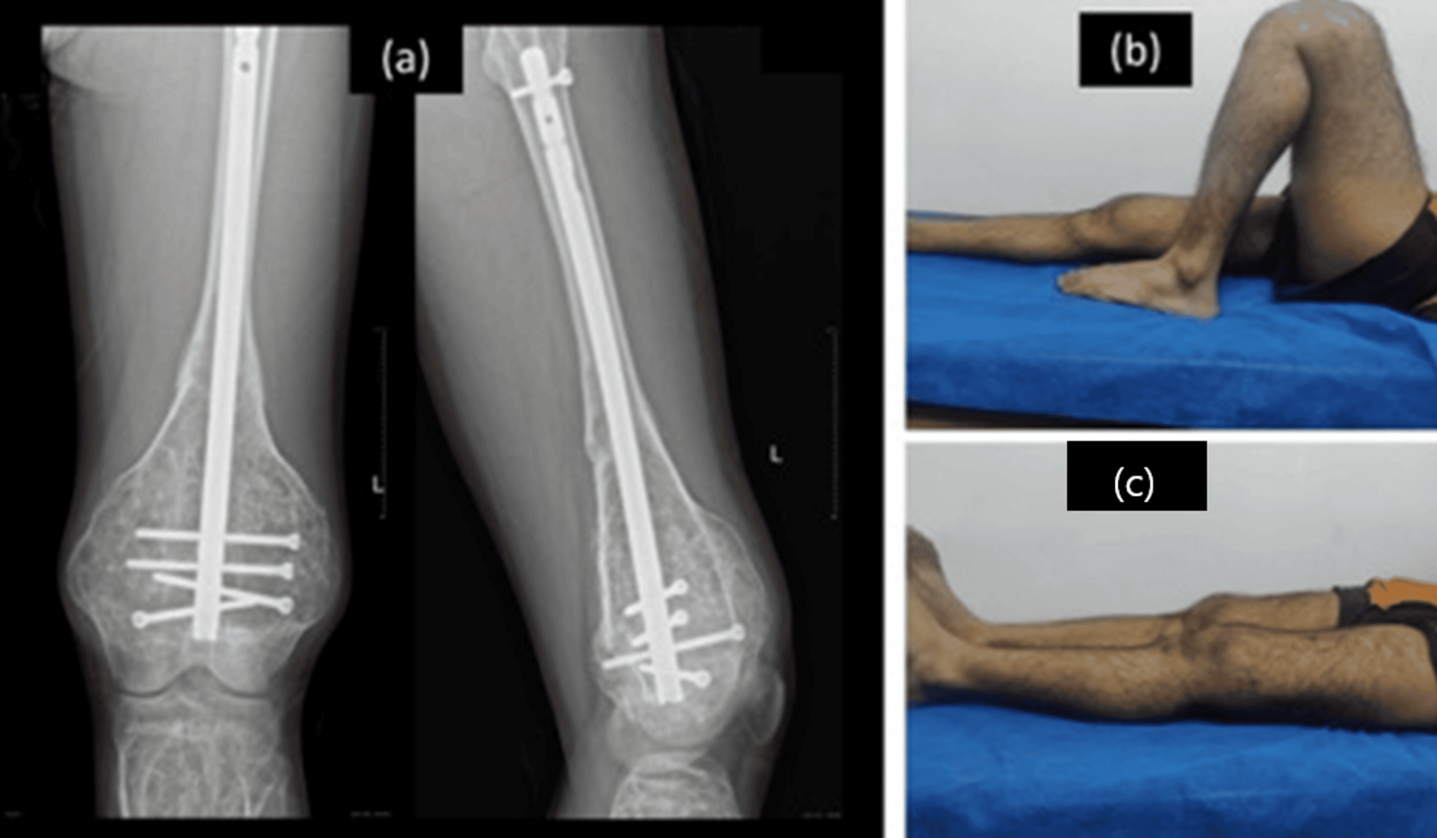
(a) Fracture union at the 3-month postoperative period; (b,c) 12-month follow-up visit-complete knee range of motion regained.

By the three-month evaluation, evidence of fracture union was observed. This union continued to progress, undergoing further remodeling and consolidation by six months. Remarkably, at this juncture, the patient had not only regained his pre-injury functional levels but also achieved a range of motion comparable to that of the uninjured side, spanning from 0° to 130°. Most importantly, the patient reported no significant complaints or limitations in the once-injured limb up to the 12-month follow-up, attesting to the successful and comprehensive resolution of the pathological fracture (Figure 7).

## Discussion

The realm of genochondromatosis remains underexplored in the annals of medical literature, with only a handful of reported cases, encompassing a mere 14 patients from five documented families [1,2,5,6]. Enchondromatosis, a complex cluster of disorders, necessitates differentiation based on lesion location, complications, and mode of inheritance [4]. Despite genochondromatosis being recognized for several years, its genetic underpinnings continue to elude complete understanding, remaining a subject of active research. Pathological fractures, a rare occurrence, have been documented in only two families, with scant details available on the management strategies employed in these cases [1,2]. Moreover, the literature addressing the management of pathological fractures in the distal femur is notably limited [8]. Recommended interventions include nailing, plating, and endoprosthesis following tumor excision. However, these approaches lack comprehensive guidance, especially concerning the challenges posed by altered patient anatomy [8,12].

In this context, our case report stands as a pioneering endeavor, presenting the first documented instance of managing a pathological distal femur fracture in a genochondromatosis patient. Our approach centered on the utilization of an intramedullary device, a strategy that offered heightened stability and the ability to span the entire femur. Adhering to the fundamental tenets of trauma fixation-maintaining length, alignment, and rotation-proved pivotal. Achieving this required meticulous pre-operative evaluation, including contralateral limb X-rays, which provided essential insights into the altered patient anatomy.

## Conclusions

This case report elucidates the role of orthopedic oncology and traumatology in the management of pathological femur fracture in genochondromatosis. The novelty lies in the decision to employ and successfully execute retrograde femoral nailing in the context of altered anatomy and flared metaphysis of the distal femur. The use of a poller pin to maneuver the nail demonstrates a nuanced adaptation of the trauma principles. The significance of this approach extends beyond genochondromatosis, underscoring the potential applicability of intramedullary devices in patients with disrupted anatomy due to benign lesions. Moreover, it holds promise in palliative case scenarios, where patients with metastatic lesions require stabilization without the excision of the tumor mass. This report provides a framework for the management of femoral fractures with dysmorphic anatomy and offers insights that can be extrapolated to similar challenges in Orthopedics.
